# Identification of Novel *Leishmania donovani* Antigens that Help Define Correlates of Vaccine-Mediated Protection in Visceral Leishmaniasis

**DOI:** 10.1371/journal.pone.0005820

**Published:** 2009-06-05

**Authors:** Sudipta Bhowmick, Nahid Ali

**Affiliations:** Infectious Diseases and Immunology Division, Indian Institute of Chemical Biology, Kolkata, West Bengal, India; Tulane National Primate Research Center, United States of America

## Abstract

Visceral leishmaniasis (VL), caused by the intracellular parasite *Leishmania donovani* is a major public health problem in the developing world. But there is no effective and safe vaccine approved for clinical use against any form of leishmaniasis. Through reactivity with kala-azar patient and cured sera, polypeptides ranging from 91 to 31-kDa from *L. donovani* promastigotes were previously identified as potential protective vaccine candidates. In this study four polypeptides 91(LD91), 72 (LD72), 51(LD51) and 31 (LD31)-kDa were purified using sodium dodecyl sulfate polyacrylamide gel electrophoresis followed by electroelution. We compared the vaccine efficacy of these antigens encapsulated in cationic liposomes in BALB/c mice against challenge infection with *L. donovani*. Our results demonstrated that liposomal LD31 (74%–77%) and LD51 (72%–75%) vaccination reduced parasite burden to the greatest degree followed by liposomal LD72 (65%–67%) and LD91 (46%–49%). Analysis of the cytokine responses in immunized mice revealed that all the vaccinated groups produced prechallenge interferon-γ, interleukin-12 and interleukin-4. Interestingly, the degree of reduction in parasite load could be predicted by the magnitude of the cytokine responses which correlated inversely with the parasite burden both in liver and spleen. The 31, 51 and 72-kDa bands were identified as ATP synthase α chain, β-tubulin and heat shock 70-related protein 1 precursor of *L. major*, respectively using matrix-assisted laser desorption ionization–time of flight (MALDI-TOF/TOF) mass spectrometry. These three leishmanial antigens have not been described before as successful vaccine candidates examined against in vivo VL model. Thus, these antigens can be potential components of future antileishmaniasis vaccines.

## Introduction

Leishmaniasis is a vector-transmitted disease distributed worldwide mainly in the tropical and subtropical countries. At least 20 *Leishmania* species can give rise to a wide spectrum of clinical manifestations, ranging from self-healing cutaneous lesions to visceral leishmaniasis (VL), the latter being an invariably fatal disease in the absence of drug treatment. Moreover, *Leishmania* is emerging as important opportunistic pathogen in persons coinfected with human immunodeficiency virus. Therapeutic options for controlling leishmaniasis are limited to a few drugs with inconsistent efficacy and side effects. Thus, there is an urgent need to develop a safe and effective *Leishmania* vaccine to help prevent the two million new cases of leishmaniasis worldwide each year [1].

Recent progress to design vaccines against leishmaniasis is based on molecularly defined antigens, the so-called second-generation vaccines. The availability of the complete *L. major* and *L. infantum* genome sequence may lead to identification of genes responsible for the different disease phenotypes [2], but vaccine candidates will probably be harder to pinpoint in the genome because without knowing the function of a pathogen protein its protective potential is not guaranteed [3]. A large number of leishmanial antigens against experimental leishmaniasis have been attempted for vaccination, mainly against the cutaneous form. There are also reports of defined antigens protective against VL in animal models such as KMP11, HASPB, A2, ORFF and CPB [4]–[8]. However there is still only a little progress and a limited number of potential candidates to combat this disease through vaccination [9]. Moreover, proteins identified from cutaneous form when tested against challenge infection with *L. donovani* in experimental models have found to be either partially protective or unsuccessful [10]–[12]. Thus there is a need for identifying new antigens from *L. donovani* ideally relying on correlates of protective immunity.

Several studies of *L. major* support that resistance towards infection is linked to the ability to mount a Th1 response which leads to the activation of macrophages for elimination of the obligatory intracellular parasite [13]. Protective immunity in *L. donovani* is also dependent on an IL-12 driven Th1 and IFN-γ production [14], [15]. However, an exclusive generation of a vaccine-induced Th1 is insufficient to ensure protection and cannot be a predictor of vaccine success in experimental VL [10], [16]. In murine cutaneous leishmaniasis (CL) a Th2 response and IL-4 production have been associated with chronic infection [13]. Although induction of IL-4 in infected BALB/c and noncuring models [17], [15] has been observed, beneficial roles of IL-4 have also been described for *L. donovani* resistance [18]. Moreover, recent studies in CL indicate that induction of high levels of the regulatory cytokine IL-10 can cause vaccine failure even in the presence of high levels of IFN-γ [19]. Thus, one of the biggest challenges for development of a vaccine against VL is to unravel the prechallenge predictor for vaccine success.

Our prior observations demonstrated that antigens of *L. donovani* promastigotes (LAg) in cationic liposomes could significantly reduce parasite load in BALB/c mice and hamsters after challenge infection [20]. We also found that LAg had strong serologic reactivity with Indian VL patient sera. While dissecting the humoral immune responses to the dominant components of LAg by Western blot analysis, we found that polypeptides with molecular masses of 31, 34, 51, 63, 72 and 91-kDa were reactive with all the kala-azar patients' sera prior to treatment. Although after successful treatment with sodium antimony gluconate (SAG) decreases in the frequency of recognition and the intensities of bands were observed, the bands were still reactive with posttreatment sera with high frequencies ranging from 73% to 100% [21]. These observations provide an insight into the vaccine potential of these antigens. Our previous report demonstrated that 63-kDa antigen entrapped in cationic liposomes induced reduction in parasite burden in BALB/c mice [22], and liposomal 34-kDa antigen was found to be ineffective to reduce parasite level in the same murine model (unpublished observation). Thus, in the present study we purified and identified the remaining four polypeptides of approximately 91, 72, 51 and 31-kDa and compared the challenge outcome with *L. donovani* and immune responses in BALB/c mice to identify key immunological correlates of protection in VL.

## Methods

### Animals and parasites

BALB/c mice, bred in the Institute animal facility (Kolkata, India), were 4–6 weeks old at the onset of the experiments. The studies were approved by IICB Animal Ethical Committee and mice were handled according to their guidelines. *L. donovani* strain AG83 (MHOM/IN/1983/AG83) promastigotes were grown at 22°C in Medium 199 supplemented with penicillin G sodium (l00 U/ml), streptomycin sulfate (100 µg/ml) and 10% heat inactivated fetal bovine serum (FBS) (Sigma-Aldrich, St. Louis, MO) and subcultured in the same medium at an average density of 2×10^6^ cells/ml [20].

### Preparation of antigens

Leishmanial antigens (LAg) were prepared from *L. donovani* promastigotes as described earlier [20]. Briefly, stationary-phase promastigotes, harvested after the third or fourth passage, were washed three times in cold 20 mM phosphate-buffered saline (PBS), pH 7.2, and resuspended at a concentration of 1.0 g of cell pellet in 50 ml of cold 5 mM Tris-HCl buffer, pH 7.6. The suspension was vortexed and centrifuged at 2,310×g for 10 min. The crude ghost membrane pellet thus obtained was resuspended in the same Tris buffer and sonicated for 3 min by ultrasound probe sonicator (Misonix, Farmingdale, NY). The suspension was centrifuged at 5,190×g for 30 min, and the supernatant containing the leishmanial antigens was harvested and stored at −70°C until use. The amount of protein obtained from a 1.0-g cell pellet, as assayed by the method of Lowry et al. [23], was approximately 10 mg.

### SDS-PAGE and electroelution

The LAg was subjected to sodium dodecyl sulfate–10% polyacrylamide gel electrophoresis (SDS–10% PAGE) by the method of Laemmli *et al.*, 1970 [24] and stained with Coomassie blue. The proteins with molecular mass of 91, 72, 51 and 31-kDa were eluted by electrophoresis in running buffer (0.025 M Tris, 0.192 M glycine, 1% SDS) using a Electro-Eluter (model 422; Bio-Rad) at 10 mA for 5 h. After elution, the proteins were dialyzed, lyophilized and resuspended in PBS. Proteins were further visualized by SDS-PAGE and silver staining [25]. The proteins were quantified by Lowry's method [23].

### Entrapment of antigens in liposomes

Cationic liposomes were prepared with distearoyl phosphatidylcholine (DSPC) (27 µmole), cholesterol (Sigma-Aldrich) and stearylamine (Fluka, Buchs, Switzerland) at a molar ratio of 7∶2∶2 as described previously [22]. Empty and antigen entrapped liposomes were prepared by the dispersion of lipid film in either 1 ml PBS alone or containing 200 µg/ml antigen respectively. The mixture was vortexed and the suspension sonicated for 30 sec by an ultrasound probe sonicator (Misonix). Liposomes with entrapped antigens were separated from excess free antigen by three successive washing in PBS with ultracentrifugation (105,000×*g*, 60 min, 4°C) [25]. The encapsulation efficiency was determined by the method of Lowry et al., in the presence of 10% SDS [23]. The level of encapsulation ranged between 50 to 60%.

### Immunization of mice and challenge infection

The experimental groups consisted of 4–6 weeks old BALB/c mice. Mice were immunized by intraperitoneal injections of 2.5 µg purified proteins in PBS or incorporated in liposome in a total volume of 200 µl. Animals receiving PBS or empty liposomes served as controls. Mice were boosted two times at 2-week intervals. Ten days after the last booster, serum samples were collected, and spleens were removed aseptically for the analysis of humoral and cellular responses after immunization. Ten days after the final immunization rest of the mice were challenged with 2.5×10^7^ freshly transformed stationary-phase promastigotes in 200 µl PBS injected intravenously via the tail vein as described earlier [20], [22]. After 3 months of challenge infection, the mice were sacrificed to determine the parasite load in liver and spleen [22]. The course of infection was monitored by the microscopic examination of Giemsa-stained impression smears of liver and spleen. The parasite load was expressed as Leishman-Donovan units and was calculated by the following formula: number of amastigotes per 1000 cell nuclei×organ weight (mg) [26].

### Assessment of delayed type hypersensitivity response (DTH)

Delayed type hypersensitivity (DTH) was determined as an index of cell mediated immunity. The response was evaluated by measuring the difference in the footpad swelling at 24 h following intradermal inoculation of the test footpad with 50 µl of LAg (800 µg/ml) and the swelling of the control (PBS injected) footpad with a constant pressure caliper (Starrett Company, Athol, MA) [20].

### Cell proliferation and cytokine assays

The spleens were aseptically removed from the immunized and infected BALB/c mice and single cell suspensions were prepared in RPMI 1640 supplemented with 10% FBS, l00 U/ml penicillin G sodium, 100 µg/ml streptomycin sulfate and 50 µM β-mercaptoethanol (Sigma-Aldrich) (complete medium). RBCs were removed by lysis with 0.14 M Tris buffered NH_4_Cl. The remaining cells were washed twice with culture medium and viable mononuclear cell number was determined by counting Trypan blue unstained cells in a hemocytometer. Then the cells were cultured in triplicate in a 96 well flat bottom plate (Nunc, Roskilde, Denmark) at a density of 2×10^5^ cells/well in a final volume of 200 µl complete medium and stimulated with antigens (2.5 µg/ml). The cells were incubated for 96 h at 37°C in a humified chamber containing 5% CO_2_. Cells were pulsed with 1 µCi of [^3^H ]-Thymidine (Amersham Biosciences, Buckinghamshire, UK) per well 18 h before they were harvested on glass fiber paper. Thymidine uptake was measured in a β-scintillation counter (Beckman Instruments, Fullerton, CA) [25]. After 72 h incubation, culture supernatants were collected and the concentration of IFN-γ, IL-4, IL-12p40 and IL-10 (BD Pharmingen, San Diego, CA) were quantitated by ELISA in accordance with the manufacture's instructions [27].

### Determination of antibody response

Sera from immunized and infected animals were analyzed by ELISA for the presence of antigen specific antibodies. In brief, 96-well microtiter plates (Maxisorp, Nunc) were coated with antigens (10 µg/ml) diluted in 20 mM phosphate buffer (pH 7.5) overnight at 4°C. The plates were blocked with 1% BSA in PBS at room temperature for 3 h to prevent nonspecific binding. After washing with PBS containing 0.05% Tween 20 (Sigma-Aldrich) (PBST) the plates were incubated overnight with 1∶1000 dilution of mice sera at 4°C. Next day, the plates were again washed with PBST and incubated further for 3 h at room temperature with horseradish peroxidase conjugated goat antimouse IgG1 and IgG2a (BD Pharmingen) diluted 1∶1000 in blocking buffer. The plates were washed and substrate solution (o-phenylene diamine dihydrochloride, 0.8 mg/ml in 0.05 M phosphate-citrate buffer, pH 5.0, containing 0.04% H_2_O_2_ ) (100 µl) was added for 30 min and the absorbance was read in an ELISA plate reader (Thermo, Waltham, MA) at 450 nm [20].

### Mass spectrometry

Protein samples were separated by SDS-PAGE and visualized with Coomassie blue or silver staining (ProteoSilver™ Plus silver stain kit, Sigma-Aldrich). The in-gel digestion of proteins was carried out according to the manufacturer's manual (Pierce, Rockford, IL). The desired band was excised and destained. The possible disulfide bonds were reduced with tris (2-carboxyethyl) phosphine (TCEP) and alkylated with iodoacetamide. Then the gel pieces were dehydrated with acetonitrile and rehydrated with 100 ng trypsin (Promega, Madison, WI) in 25 mM ammonium bicarbonate solution and were incubated at 37°C overnight. The tryptic fragments were extracted from the gel by adding 1% trifluroacetic acid. Then, the peptides were purified with C18 reversed-phase minicolumn filled in a micropipette tip, ZipTip C18 (Millipore, Bedford, MA). Purified peptides (0.5 µl) were cocrystallized with α-cyano-4-hydroxy cinnamic acid matrix (0.5 µl) (Applied Biosystems, Foster City, CA) on a matrix-assisted laser desorption ionization (MALDI) target plate. Both mass spectrometry (MS) and MS/MS spectra were acquired by matrix-assisted laser desorption ionization–time of flight (MALDI-TOF/TOF) Mass Spectrometer (Applied Biosystems 4800 Proteomics Analyzer). All spectra were collected in the reflector mode. Calibration was updated before each acquisition using a standard peptide mixture according to instrument protocol. Database searching for protein identification was performed with mass spectrometry data using GPS Explorer (Applied Biosystems) software with MASCOT (Matrix Science) search engine.

### N-terminal sequencing

The antigens were subjected to SDS-PAGE electrophoresis, and electrotransferred onto a polyvinylidene difluoride membrane (Immobilon P; Millipore) in 10 mM 3-[cyclohexylamino]-1-propanesulfonic acid and 10% methanol, pH 11.0. The bands on the membrane were excised after staining according to manufacturer's instructions. The N-terminal amino acid sequence was determined by Edman degradation chemistry using a Procise 492 Protein sequencer (Applied Biosystems) [22].

### Statistical analysis

One-way analysis of variance (ANOVA) and Tukey's multiple comparisons post-test were used for the analysis of parasite burden and antibody response data using Graph Pad Prism version 4.0 (Graphpad Software, v. 4.0, San Diego, CA). The statistical analyses for other experiments before and after infection were made by a two-way ANOVA. Corrections to the level of significance were done using Bonferroni's method. Results with p<0.05 were considered to be statistically significant. For correlation analysis, all datasets were calculated for correlation efficiency and were considered significant at p<0.05. Spearman's rank correlation coefficient values were evaluated and represented (r).

## Results

### Parasite burdens in vaccinated BALB/c mice after *L. donovani* challenge infection

**Figure 1:**
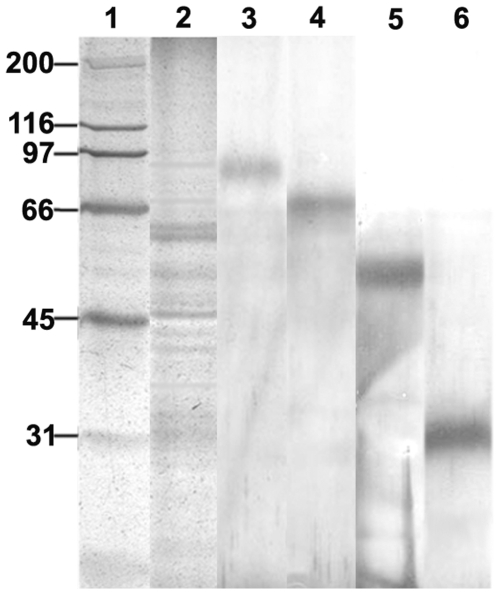
Silver-stained SDS-PAGE gel of Leishmanial antigens (LAg) and purified antigens by electroelution. Lane 1, molecular mass markers; lane 2, LAg ; lane 3, 91-kDa (LD91); lane 4, 72-kDa (LD72); lane 5, 51-kDa (LD51); lane 6, 31-kDa (LD31). The positions of molecular mass standards are shown on the left in kilodaltons.

**Figure 2:**
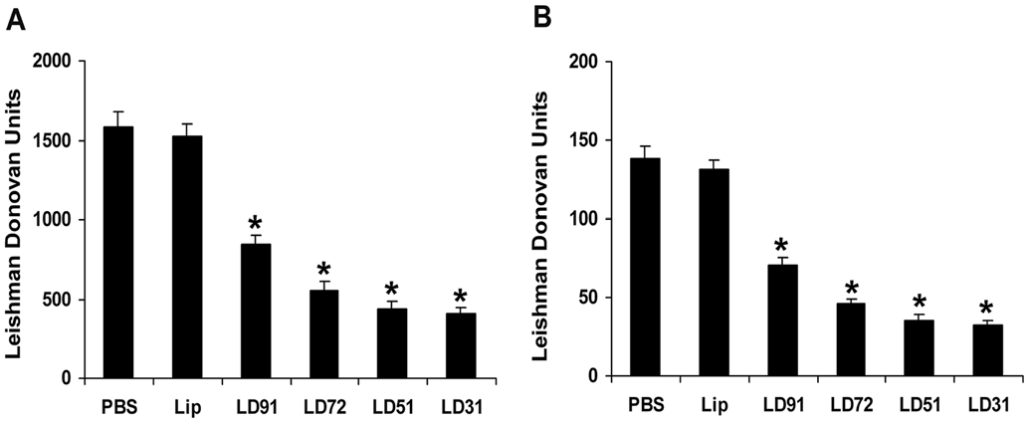
Parasite burdens in BALB/c mice vaccinated with cationic liposomal antigens after *L. donovani* challenge infection. Mice were vaccinated intraperitoneally 3 times with 2.5 µg of each antigen entrapped in cationic liposomes at 2-week intervals. Control groups received PBS or empty liposomes (Lip). At 10 days after the last immunization, the mice were challenged intravenously with 2.5×10^7^ promastigotes of *L. donovani.* Liver (A) and spleen (B) parasite burden were measured 3 months after challenge as Leishman Donovan Units (LDU). The results are mean LDU±S.E. of five individual mice per group, representative of two independent experiments with similar results. * p<0.001 in comparison to control groups as assessed by one-way ANOVA and Tukey's multiple comparison test.

Proteins from LAg ranging from 91 to 31-kDa were purified by eletroelution from 10% SDS-PAGE gel and then visualized on SDS-PAGE followed with silver staining (Figure 1). BALB/c mice were immunized intraperitoneally with antigens LD91 (91-kDa), LD72 (72-kDa), LD51 (51-kDa) and LD31 (31-kDa) entrapped in cationic liposomes as the adjuvant. The vaccination was repeated twice at 2-week intervals and the mice were challenged intravenously with L. donovani promastigotes 10 days after the last immunization. Control mice were injected with the empty liposomes or PBS. We and others have found that infection with the Indian strain, L. donovani AG83 in BALB/c mice results in a progressive infection in the liver and spleen, corresponding with hepato and splenomegaly [22], [27]–[30]. Mice were therefore sacrificed after 3 months when parasite loads were well expressed in both liver and spleen. In liver, the greatest degree of reduction in parasite burden was observed in mice immunized with liposomal LD31 (74%) followed by LD51 (72%) (Figure 2A). Although immunization with liposomal LD31 induced higher levels of reduction than liposomal LD51, the difference was not statistically significant. Vaccination with liposomal LD72 (65%) conferred reduction in parasite load to a moderate level. Immunization with LD91 in liposomes (46%) also reduced parasite load significantly in mice but to the lowest degree (p<0.001 compared to controls). In BALB/c mice persistence of L. donovani in the spleen causes concomitant development of considerable organ-specific pathology similar to that seen in the human kala-azar. It was, therefore, important to evaluate the effect of vaccination in this organ. Similar to liver, mice immunized with liposomal LD31 (77%) and LD51 (75%) demonstrated highest reduction in splenic parasite burden with a moderate level in mice immunized with LD72 (67%) and lowest but significant level with LD91 (49%, p<0.001 compared to controls) (Figure 2B). It may be noted that the reduction in parasite burden by the different liposomal antigens were maintained in both the organs.

**Figure 3:**
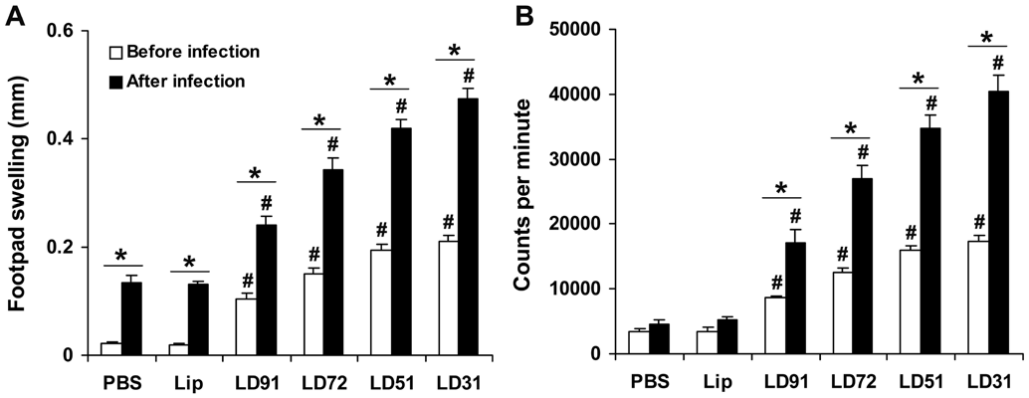
DTH and splenocyte proliferation in BALB/c mice vaccinated with cationic liposomal antigens before and after challenge infection. Ten days after the last vaccination and 3 months after challenge infection DTH (A) and splenocyte proliferative (B) responses were measured. DTH response is expressed as the difference (in millimeters) between the thickness of the test (LAg-injected) and control (PBS-injected) footpads at 24 h. Spleens were collected and splenocytes were restimulated in vitro for 72 h with 2.5 µg/ml of specific antigens. Antigen-specific splenocyte proliferation was determined by thymidine incorporation and expressed as counts per minute. Each sample was examined in triplicate. The results are shown as the mean±S.E. for five individual mice per group, representative of two independent experiments with similar results. # p<0.0001 in comparison to control groups and * p<0.001 in comparison to data before infection as assessed by two-way ANOVA and Bonferroni's correction.

DTH and splenocyte proliferative responses in vaccinated mice before and after challenge infection DTH, an index of cell-mediated immunity in vivo, and antigen induced proliferation of splenocytes in vitro were evaluated in different vaccinated mice 10 days after the last immunization. Among the different vaccinated groups, liposomal LD31 and LD51 immunized groups exhibited the highest degree of both DTH (Figure 3A, p<0.0001) and proliferative response of splenocytes (Figure 3B, p<0.0001) compared to controls. Liposomal LD72 and LD91 also induced considerably higher responses than controls (p<0.0001). Controls exhibited almost similar level of splenocyte proliferation after stimulation with different antigens. Stimulation with total LAg also enhanced the proliferative response of the vaccinated groups but lower in magnitude as observed with specific antigens (data not shown). The responses were further measured after 3 months of L. donovani infection. At this stage, all the groups of mice presented increased DTH and proliferative responses, compared to their respective levels before infection (p<0.001). Similar to prechallenge, mice immunized with all four liposomal LD31, LD51, LD72 and LD91 displayed enhanced but varying magnitudes of both DTH and antigen-induced splenocytes proliferative responses (p<0.0001 compared to controls) at 3 months post infection.

### Cytokine responses in vaccinated mice before and after challenge with *L. donovani*

**Figure 4:**
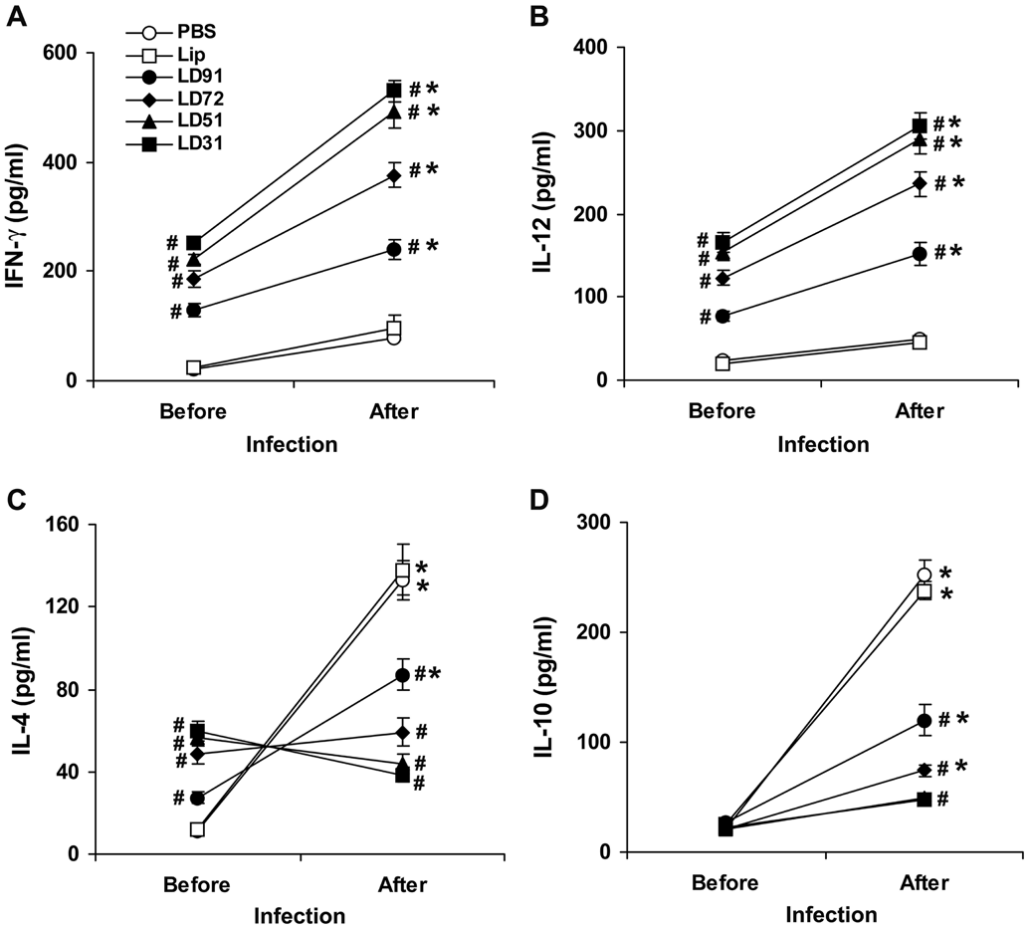
Cytokine responses in BALB/c mice vaccinated with cationic liposomal antigens before and after challenge infection. Ten days after the last vaccination and 3 months after challenge infection spleens were collected and splenocytes were restimulated in vitro with 2.5 µg/ml of specific antigens. After 72 h supernatants were collected and assayed for IFN-γ (A), IL-12 (B), IL-4 (C) and IL-10 (D) levels by ELISA. Each sample was examined in duplicate. The results are shown as the mean±S.E. for five individual mice per group, representative of two independent experiments with similar results. # p<0.0001 in comparison to control groups and * p<0.001 in comparison to data before infection as assessed by two-way ANOVA and Bonferroni's correction.

Next, we analyzed different cytokine production by splenocyte following restimulation with specific antigens in vaccinated mice. Following immunization, different vaccinated groups produced substantial amounts of IFN-γ (Figure 4A), IL-12 (Figure 4B) and IL-4 (Figure 4C) compared to controls (p<0.0001). There was no difference in the cytokine production in control animals when stimulated in vitro with different antigens. Both liposomal LD31 and LD51 secreted highest level of IFN-γ and IL-12 compared to controls (p<0.0001). Interestingly, these groups also secreted IL-4 in high magnitude (p<0.0001 compared to controls). Before infection there was no stimulation of IL-10 production in any of the vaccinated groups in response to antigens (Figure 4D). Splenocytes of different vaccinated mice also secreted increased amounts of IFN-γ, IL-12 and IL-4 when stimulated with total LAg but response was lower than with the specific antigens (data not shown). Taken together, the quality of cytokine responses in different vaccine groups represented production of IFN-γ, IL-12 and IL-4 before infection which correlated with the decreased parasite level in post infection. In addition, these results suggested a link between the magnitude of these cytokine responses and the extent of reduction in parasite burden.

With progressive infection, levels of IFN-γ and IL-12 remained low although pronounced stimulation of IL-4 and IL-10 (p<0.001) compared to the level before infection, were observed in the supernatants from splenocytes of control infected mice stimulated with different antigens. Splenocytes from mice immunized with liposomal LD31, LD51 and LD72 secreted high levels of IFN-γ and IL-12 (p<0.0001 compared to controls) significantly enhanced in comparison to prechallenge (p<0.001). Levels of IL-4 and IL-10 remaind low (p<0.0001 compared to controls) in these vaccinated groups with decreased parasite load. In contrast, IFN-γ and IL-12 levels were comparatively lower in LD91 immunized mice with higher levels of IL-4 and IL-10 production. An increase, however was observed in the levels of all the cytokines when compared to prechallenge response (p<0.001).

### Production of IgG2a and IgG1 before infection is predictive of successful vaccination

**Figure 5:**
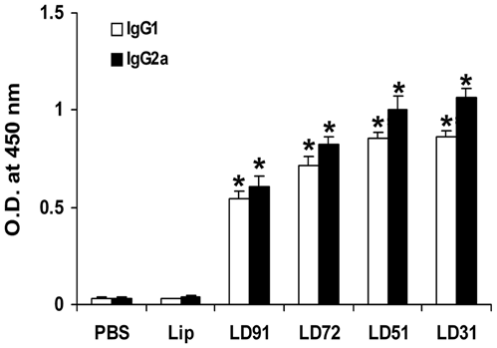
Prechallenge antigen-specific IgG isotype responses in BALB/c mice vaccinated with cationic liposomal antigens. Ten days after last vaccination serum samples were collected and assayed for antigen-specific IgG1 and IgG2a antibodies by ELISA. Each sample was examined in duplicate. The results are shown as the mean absorbance values±S.E. of five individual mice per group, representative of two independent experiments with similar results. * p<0.001 in comparison to control groups as assessed by one-way ANOVA and Tukey's multiple comparison test.

Because cytokines such as IFN-γ and IL-4 direct immunoglobin class switching for IgG2a and IgG1, respectively [31] we measured antigen specific production of these antibody isotypes before infection to provide an indirect but physiologic in vivo assessment of the pattern of cytokine production and consequently reduction of parasite load after challenge infection. Sera from mice immunized with liposomal LD31 and LD51 contained elevated and highest levels of both the IgG2a and IgG1 isotypes (Figure 5, p<0.001 compared to controls), although the level of IgG2a was higher than that of IgG1. Significant enhancement for both the isotypes with a dominance of IgG2a were also observed in liposomal LD72 immunized groups with minimum production of the isotypes in liposomal LD91 group (p<0.001 compared to controls) These results further substantiate the magnitude of the production of both the cytokines, IFN-γ and IL-4, before infection is predictive of success of vaccination.

### Assesment of immune correlates of protective immunity

**Figure 6:**
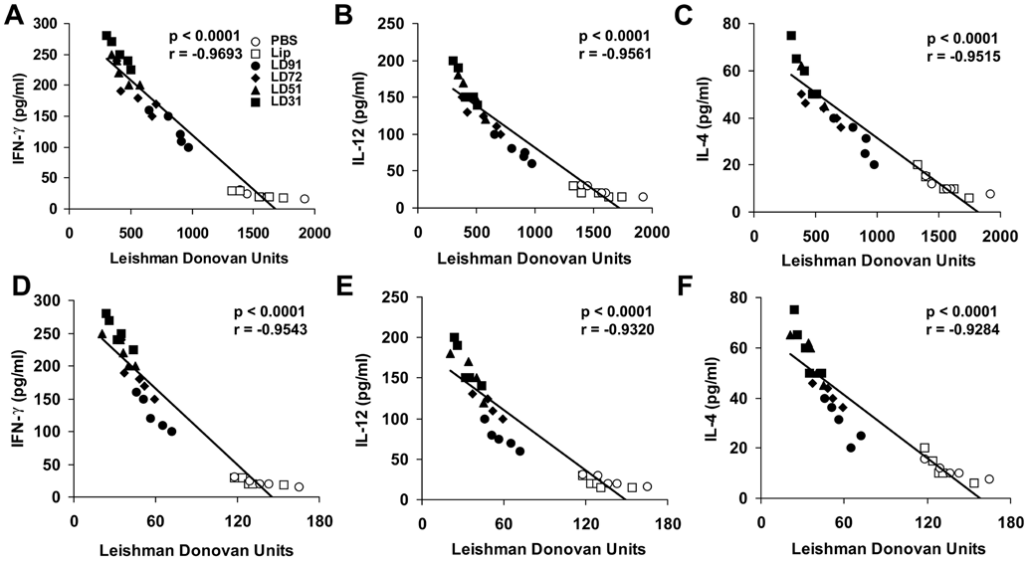
Correlates of immune responses and reduced parasite burdens in BALB/c mice vaccinated with cationic liposomal antigens. Liver and spleen parasite burden after 3 months challenge infection with *L. donovani* promastigotes was tested for correlation with antigen-specific prechallenge IFN-γ, IL-12 and IL-4 responses. Magnitudes of the antigen-specific IFN-γ, IL-12 and IL-4 production were inversely correlated with the liver (A to C) and spleen (D to F) parasite burdens. Each symbol represents an individual animal from a vaccinated group. The p-values were calculated for correlation efficiency and were considered significant at p<0.05. Spearman's rank correlation coefficient values are represented (r). Diagonal lines represent linear regression.

Differences in the IFN-γ, IL-12 and IL-4 production between vaccine groups prompted us to evaluate the potential correlations between the cytokine responses after immunization and parasite burden in the vaccinated animals of the representative groups. Importantly there was a strong inverse correlation between the liver parasite burden and magnitude of the IFN-γ production before infection (Figure 6A). Also, the differential production of IL-12 (Figure 6B) and IL-4 (Figure 6C) correlated with control of infection, suggesting the identification of associates of reduced parasite burden after immunization. A statistical correlation between the cytokine responses and pathogenesis was then determined in spleen. There was also a striking inverse correlation between the IFN-γ, IL-12 and IL-4 (Figure 6D to F) productions and the parasite burden in spleen, signifying a direct correlation of parasite burden with the magnitude of the cytokine responses after immunization.

### Identification of antigens which conferred protection against challenge infection

Immunization with liposomal LD31, LD51 and LD72 exhibited enhanced reduction in parasite burden with a preferable immune response against L. donovani challenge. To identify these candidate vaccine antigens, the bands were excised, subjected to in-gel trypsin digestion. The digested proteins were analyzed by MALDI-TOF/TOF mass spectrometry, and the MS and MS/MS spectrum were searched against all the known Leishmania spp. sequences in MSDB and NCBInr databases using MASCOT search engine. LD31 was identified as ATP synthase α chain of L. major (Table 1). The band of LD51 was identified as β-tubulin of L. major. Again, the N-terminal sequence of LD51 found to be MREIVGCQAGQCGNQ, which corresponds to the β-tubulin of L. major and L. donovani. Mass spectrometry of trypsin digested LD72 was also identified as heat shock 70-related protein 1 mitochondrial precursor of L. major. Interestingly, the tryptic digests of LD91 demonstrated the presence of β-tubulin of L. major with 6 matched peptides.

**Table 1:** Proteins identified by MALDI-TOF/TOF mass spectrometry.

Band	Protein name	Accession Number^a^	Matching Peptides	Probability-based Mowse score	% Sequence coverage
LD31	ATP synthase α chain (*L. major*)	68223738	18	324	20
LD51	β-tubulin (*L. major*)	68224035	18	223	27
LD72	Heat shock 70-related protein 1 mitochondrial precursor ( *L. major*)	57015345	11	71	18
LD91	β-tubulin (*L. major*)	68224035	6	57	14

a Accession number in NCBInr database.

## Discussion

Over the past years major efforts have been dedicated to develop novel vaccine candidates against leishmaniasis and evaluate them in a variety of animal models. These efforts were fueled by the publication of the *L. major* and *L. infantum* genome and renewed interest in the field of leishmaniasis prophylaxis led to the identification of a plethora of putative candidates for vaccine development [2]. The vaccine components identified from genome however, requires critical in vivo experimental evaluation to establish their utility as candidate antigens. Although recombinant proteins fulfill the key criteria, like consistent product quality and cost-effectiveness, studies involving native proteins rather than recombinant ones are needed, as the latter may lack similar immunogenic characteristics [32]. Several vaccine candidates have been identified and evaluated against different *Leishmania* spp. However, in spite of homology between the different species and a certain degree of cross protection, the need for identification of new immunostimulatory molecules from individual species is still required. Additionally, the immune response that mediate susceptibility or resistant to *L. donovani* infection differ significantly from other species of *Leshmania*, especially *L. major*. In CL a polarized Th1 response is sufficient for protection and a concomitant Th2 abrogates even a strong Th1 function to favor the disease progression. However, this Th1/Th2 dichotomy is lacking in VL and a mixed response is essential for protection [6], [13], [16], [33]. But the immune parameters, which were required to predict protection in VL, are still unresolved.

Although cell mediated immune responses are thought to play important role in controlling leishmaniasis, ability to induce Th1 response might not be the only criteria to select vaccine targets against VL [10], [34]. Screening of expression libraries with sera from infected animals and humans have been widely used to identify most immunogenic novel vaccine antigens against leishmaniasis [35]. As individuals with acute VL develop a polyclonal B-cell response generating antibodies to a large number of parasite antigens, a two-step library screening procedure first by reactivity with patients' sera followed by their ability to induce T cell proliferation and IFN-γ responses in immune mice might be more useful [36]. Subsequently, antigens such as HASPB1 and A2 that induce strong protection against VL also showed serologic reactivity with patients' sera [37], [38]. In our attempt to design a vaccine against VL, we used a different approach and selected four antigens that exhibited humoral reactivity with not only sera of infected VL patients but also demonstrated persistence recognition with cured sera. Moreover, PBMC from VL patients showed proliferative response with an elevation in IFN-γ and IL-12 production when stimulated with these antigens (unpublished observation). Thus, in the absence of an efficient method to predict protective antigens, we then critically evaluated the vaccine efficacy of these antigens individually using an in vivo experimental mice model against *L. donovani* challenge infection.

The findings in this report demonstrated that vaccination with these polypeptides entrapped in cationic liposomes conferred reduction in parasite burden in BALB/c mice against visceral infection with *L. donovani*. Experiments performed herein also demonstrated that stimulation of three cytokines, IFN-γ, IL-12 and IL-4, before infection correlated with decreased parasite level. All the vaccinated groups produced these three prechallenge cytokines suggesting this quality of response is required for successful vaccination against VL. We also observed a direct correlation between the magnitude of the response and the level of reduction in parasite load. Liposomal LD31 and LD51 immunization exihibited highest level of these three cytokines, reduced parasite level in mice to the greatest degree. Immunization with LD72 with moderate response led to a lower level of reduction whereas liposomal LD91 immunization induced lowest level of these cytokines and reduction of parasite load. Thus, not only the quality but also the magnitudes of the prechallenge cytokine responses were critical correlates of protection in VL. The findings in this report with subunit vaccines also substantiate our earlier observation with leishmanial antigens that an early mixed Th1/Th2 response was required for success against VL [27], [29]. Significantly elevated levels of IgG2a antibody along with increased levels of IgG1 in vaccinated groups before infection observed herein suggested a mixed Th1/Th2 cytokine profile. Again the magnitude of the antibody response correlated with the degree of reduction of parasite burden further supporting the implication of this immune correlate.

The quality and magnitude of the immune response may be regulated by the synergistic effect of these three cytokines. An IL-12 driven IFN-γ-dependent Th1 response associated with protective immunity, has a direct effect on the macrophage microbicidal activity and other effector killing mechanisms [13]. IL-12 is known to be involved not only in activation of NK cell but also development of Th1 response and granuloma formation. Moreover, it may have important role to establish cell-mediated immunity by promoting the differentiation of Th1 cells from naive T cells and also to maintain that immunity [39]. The essential role of IL-12 has been confirmed in vivo by the treatment of mice with recombinant IL-12 at the time of infection that shifted the immune response to Th1 and prevented susceptibility to infection [40]. Additionally, immunization with a combination of leishmanial antigens and IL-12 led to the development of a Th1 response and conferred protection in BALB/c mice [41]. Another important candidate is IL-4, a known Th2 cytokine. Previous reports have shown that IL-4 is needed to drive Th1 differentiation [42], to maintain IFN-γ production [43] and to prime IL-12 production [44]. This suggests that an early IL-4 may have a role in an initial induction of protective immunity. The finding that IL-4^−^/^−^ knockout mice were more susceptible to *L. donovani* [18] and the reported beneficial role of IL-4 in effective chemotherapy [45], suggest the protective ability of this cytokine in VL. Thus, combinatorial effects of these cytokines cause an additional paramount effect on magnitude of the protective immune response. None of the protected groups produced IL-10 after immunization, suggesting that IL-10 has no role in protection. It is hypothesized that production of IL-10 may be a prechallenge indicator for vaccine failure in CL [19], and thus absence of IL-10 after immunization may also have beneficial role in VL for protection.

As shown in this study LD31, LD51 and LD72 are protective vaccine antigens, we had identified the proteins by MALDI-TOF and N-terminal sequencing. The success of this approach was made possible due to the availability of the entire sequence of *L. major* and *L. infantum* genome. 31-kDa polypeptide was identified as ATP synthase α chain of *L. major*. 51-kDa band was identified as β-tubulin and 72-kDa was identified as heat shock 70-related protein 1 precursor of *L. major*. To our knowledge vaccine potentiality of two of these antigens ATP synthase and β-tubulin have never been evaluated against any form of leishmaniasis. *L. major* HSP70 was evaluated as recombinant protein but failed to induce protection against *L. major* [46].

ATP synthase complex subunits are functionally associated with the membrane and thus can be potential targets for drugs, diagnostic probes or vaccine components against *Leishmania*. ATP synthase α subunit was already identified as immunogenic proteins in *Mycobacterium bovis* [47] and intracellular pathogens like *Brucella* [48] when probed with antisera from bovine and human patient. The discrepancy in the observed and predicted molecular weight, which seems to be 62-kDa, suggests a probable truncated form of this protein. This phenomenon has been widely reported in the proteomes of several *Leishmania* spp. as well as in *Trypanosoma* [49], [50]. Moreover, for membrane proteins often the gel molecular weights do not correspond to the actual molecular weights based on amino acid composition and bands may migrate anomalously in SDS-PAGE [51]. Additionally, the difference in observed mass from that predicted by the leishmanial genome, a common feature reported in most proteomic analyses, may be the effect of protein ‘maturation’ events including co- or post-translational modifications (PTM). The observations from other studies of *Leishmania* suggest that PTM is widely prevalent in this organism [49], [50]. 51-kDa band, identified as β-tubulin has previously been characterized as a T-cell stimulating antigen from *Leishmania* by CD4^+^ T cell expression cloning [52]. Moreover, tubulin, has also been identified as a vaccine candidate from a phage expression library using sera of VL patients [53]. The 91-kDa polypeptide with a homology with β-tubulin did not show immunogenicity and protective immunity to that level as observed with 51-kDa antigen. This 91-kDa band suggested that PTMs are the most likely cause of variant forms of tubulin [49], [50]. There are several different types of modifications like methylation and mainly glycosylation which include fucosylation and hexosylation, that are common to β-tubulin [54]. Such multiple isoforms of tubulin caused by PTMs were common in *Leishmania* and *Trypanosoma* [49], [50]. As a result in a proteomic study with *T. brucei* 84 gel spots were predicted to match with β-tubulin [50]. *Leishmania* HSP70 are known to potentiate a Thl-type response and can act as an adjuvant when coconstruced with DNA vaccine [55]. Although immunization with *L. major* HSP70 failed to induce protective responses in CL [46], this antigen produce a Th1-type immune response, and was identified as a probable vaccine candidate for canine VL [56]. The microbial HSP70s have acquired special significance in immunity since they have been shown to be potent activators of the innate immune system via toll like receptor (TLR)-2 and TLR-4 signalling [57].

Although tubulin and HSP70 are highly conserved molecules, there are still sufficient levels of heterogeneity between the parasite and the host. In β-tubulin, despite 86% homology to its human counterpart, the parasite tubulin-specific T cell reactivity was observed in CL patients and no response was elicited by the purified mammalian tubulin. Epitope mapping experiments also showed that T cell reactivity was restricted to a small block of nonconservative amino acid substitutions, suggesting subtle sequence differences between the proteins of the invading pathogen and the corresponding host homologs [52]. Similarly, recombinant *Leishmania* HSP70 was effective in stimulating PBMC from *L. braziliensis*-infected individuals to proliferate and produced IFN-γ [58]. But PBMC from mucosal patients were not stimulated by recombinant human HSP70 to either proliferate or produce cytokines despite the fact that *Leishmania* and human hsp70s share 72% homology [58]. In our study interestingly, during MALDI-TOF data analysis these proteins were identified as protein of *L. major* during the first search, when no restriction was applied to the species of origin. Moreover, recognition of these proteins by the sera from *L. donovani* infected patients indicated that there are amino acid differences between *Leishmania* and host proteins that are sufficiently distinct to allow recognition of the leishmanial protein by the immune system.

In conclusion, our study depicts that optimal reduction of parasite burden in VL requires stimulation of prechallenge IFN-γ, IL-12 and IL-4 following vaccination. Success against VL depends on the synergistic effect of these three cytokines as well as their magnitude, and may vary depending on the vaccines. Further, identification of three successful vaccine antigens indicates that these proteins in their recombinant form can potentially be putative candidates of a subunit vaccine against VL.
